# Prevalence and Diversity of Small Mammal-Associated *Bartonella* Species in Rural and Urban Kenya

**DOI:** 10.1371/journal.pntd.0003608

**Published:** 2015-03-17

**Authors:** Jo E. B. Halliday, Darryn L. Knobel, Bernard Agwanda, Ying Bai, Robert F. Breiman, Sarah Cleaveland, M. Kariuki Njenga, Michael Kosoy

**Affiliations:** 1 Boyd Orr Centre for Population and Ecosystem Health, Institute of Biodiversity, Animal Health and Comparative Medicine, College of Medical, Veterinary and Life Sciences, University of Glasgow, Glasgow, United Kingdom; 2 Center for Conservation Medicine and Ecosystem Health, Ross University School of Veterinary Medicine, Basseterre, St. Kitts; 3 National Museum of Kenya, Nairobi, Kenya; 4 Centers for Disease Control and Prevention, Division of Vector-Borne Diseases, Fort Collins, Colorado, United States of America; 5 Division of Global Health Protection, Atlanta, Georgia, United States of America; 6 Emory Global Health Institute, Emory University, Atlanta, Georgia, United States of America; 7 Kenya Medical Research Institute/CDC Public Health and Research Collaboration, Kisumu and Nairobi, Kenya; 8 Global Disease Detection Division, CDC-Kenya, Nairobi, Kenya; Institute of Parasitology, AUSTRIA

## Abstract

Several rodent-associated *Bartonella* species are human pathogens but little is known about their epidemiology. We trapped rodents and shrews around human habitations at two sites in Kenya (rural Asembo and urban Kibera) to determine the prevalence of *Bartonella* infection. *Bartonella* were detected by culture in five of seven host species. In Kibera, 60% of *Rattus rattus* were positive, as compared to 13% in Asembo. *Bartonella* were also detected in *C*. *olivieri* (7%), *Lemniscomys striatus* (50%), *Mastomys natalensis* (43%) and *R*. *norvegicus* (50%). Partial sequencing of the citrate synthase (gltA) gene of isolates showed that Kibera strains were similar to reference isolates from *Rattus* trapped in Asia, America, and Europe, but that most strains from Asembo were less similar. Host species and trapping location were associated with differences in infection status but there was no evidence of associations between host age or sex and infection status. Acute febrile illness occurs at high incidence in both Asembo and Kibera but the etiology of many of these illnesses is unknown. *Bartonella* similar to known human pathogens were detected in small mammals at both sites and investigation of the ecological determinants of host infection status and of the public health significance of *Bartonella* infections at these locations is warranted.

## Author Summary

*Bartonella* are bacteria that infect many different mammal species and can cause illness in people. Several *Bartonella* species carried by rodents cause disease in humans but little is known about their distribution or the importance of bartonellosis as a cause of human illness. Data from Africa are particularly scarce. This study involved trapping of rodents and other small mammals at two sites in Kenya: Asembo, a rural area in Western Kenya, and Kibera, an informal urban settlement in Nairobi. Blood samples were collected from trapped animals to detect and characterize the types of *Bartonella* carried. At the Kibera site over half of the trapped rats were infected with *Bartonella* very similar to human pathogenic strains isolated from rats from other global regions. In Asembo, *Bartonella* were detected in four of the five animal species trapped and these *Bartonella* were less similar to previously identified isolates. All of the small mammals included in this study were trapped in or around human habitations. The data from this study show that *Bartonella* that can cause human illness are carried by the small mammals at these two sites and indicate that the public health impacts of human bartonellosis should be investigated.

## Introduction

*Bartonella* species are Gram-negative haemotrophic bacteria that infect mammalian erythrocytes and are transmitted between hosts by blood-sucking arthropods. Over 30 species of *Bartonella* have been described and members of this genus infect a broad range of mammalian hosts including rodents, bats, carnivores and ruminants [1]. Arthropod vectors including fleas, sandflies, lice, ticks, bat flies and ked flies are implicated in the transmission of these pathogens [2–4]. The genus *Bartonella* has a global distribution. The *Bartonella elizabethae* complex includes several *Bartonella* genotypes and strains (including *B*. *elizabethae*, *B*. *tribocorum*, *B*. *rattimassiliensis* and *B*. *queenslandensis*) that have been isolated from *Rattus* and *Bandicota* species around the world [1]. Recent analyses indicate that this complex has south-east Asian origins and has been globally dispersed by *Rattus* species [5].

Several *Bartonella* species are recognized as human pathogens that cause diverse clinical presentations [6]. Among rodent-associated *Bartonella* species, *B*. *elizabethae* is a known cause of human endocarditis [7]. Other rodent-associated species including *B*. *tribocorum*, *B*. *vinsonii subsp*. *arupensis*, *B*. *washoensis* and *B*. *alsatica* have been associated with a range of symptoms in humans including fatigue, muscle and joint pain, and serious complications, such as endocarditis and neurological signs, particularly in immunocompromised patients [8,9].

*Bartonella* species have been identified as important causes of febrile illness in some settings. In two studies conducted in Thailand, 15% of febrile patients were diagnosed with confirmed *Bartonella* infection based on a four-fold rise in antibody titres, and six different *Bartonella* species were identified by culture from blood clots collected from febrile patients [10,11]. Non-specific clinical signs and difficulties in culturing the organism present substantial challenges to the diagnosis of bartonellosis. Consequently, *Bartonella* species may well be under-recognized as a cause of human disease [12]. This is particularly true for Africa, where very few data on the etiology of febrile illness are currently available [13].

In the Democratic Republic of Congo (DRC), a seroprevalence study identified IgG antibodies against *Bartonella* (*B*. *henselae*, *B*. *quintana* or *B*. *clarridgeiae*) in 4.5% of febrile patients [14]. *Bartonella* bacteraemia was detected by PCR in 10% of HIV-positive patients in South Africa [15]. Apart from these studies however, there is little information on the impact of *Bartonella* on human health on the African continent.

A variety of *Bartonella* species have been detected in animal and ectoparasite populations in Africa. Considering rodents and small mammals specifically, *B*. *elizabethae* and two other *Bartonella* lineages were detected in Namaqua rock mice sampled in South Africa, where 44% of the 100 individuals sampled were positive by PCR for *Bartonella* species [16]. *B*. *elizabethae*, *B*. *tribocorum* and a *Bartonella* species with intermediate species classification based on sequence data were detected in 28% of rodents and hedgehogs (n = 75) sampled in Algeria [17]. *B*. *elizabethae*, *B*. *tribocorum* and novel *Bartonella* species were also detected in rodents sampled in the Democratic Republic of Congo (DRC) and Tanzania [18]. Small mammals trapped in Ethiopia, had an overall *Bartonella* infection prevalence of 34% (n = 529) and were infected with multiple genotypes including genotypes very closely related to *B*. *elizabethae* [19]. *B*. *elizabethae* has also been detected in invasive and indigenous rodents sampled in Uganda [20] and genotypes related to *B*. *rochalimae*, *B*. *grahamii* and *B*. *elizabethae* have been detected in Mearn’s pouched mice studied in Kenya [21]. *Bartonella* have also been detected in fleas collected in Egypt, Morocco, DRC and Uganda [20,22–24].

The first objective of this study was to determine the presence and prevalence of *Bartonella* infections in small mammals trapped at rural and urban locations in Kenya. We also aimed to characterize the *Bartonella* isolates obtained using partial sequences of the citrate synthase (*gltA*) gene and to compare the *Bartonella* genotypes detected in these distinct Kenyan populations with each other and with *Bartonella* detected in small mammals in other parts of the world.

## Materials and Methods

### Study sites

**Fig 1:**
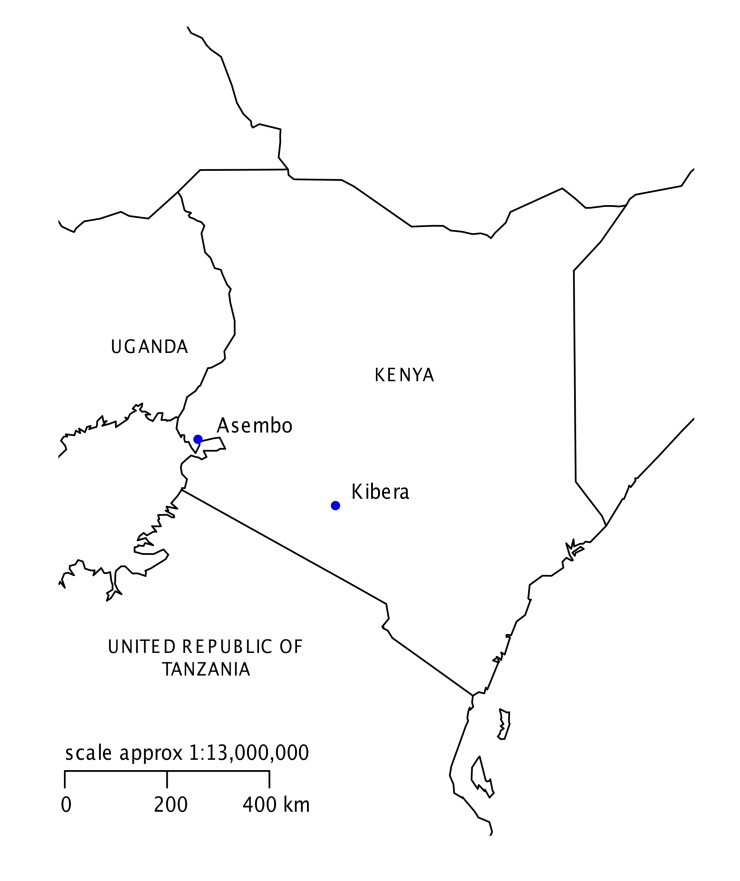
Map of Kenya indicating the location of study sites.

Cross-sectional rodent trapping surveys were conducted within two locations: Asembo, a rural area on the northern shore of Lake Victoria in Nyanza Province western Kenya (Latitude-0.1443, longitude 34.3468) and Kibera, an urban informal settlement in Nairobi City (Latitude-1.3156, longitude 36.7820, Fig. 1). These locations are the study sites for ongoing population-based human health surveillance [25]. In Asembo, subsistence farming is the primary occupation for 65% of household heads, 13% work in the informal economy and 5% are salaried [25]. Households are clustered into compounds of closely related family units. Livestock ownership is common: 44% of Asembo households own cattle and 43% own at least one sheep or goat. In contrast, in urban Kibera, 53% of heads of household are salaried and 43% work in the informal sector [25]. Ownership of large livestock species in Kibera is very rare and prohibited by City Council law.

### Small mammal trapping and sample collection

**Fig 2:**
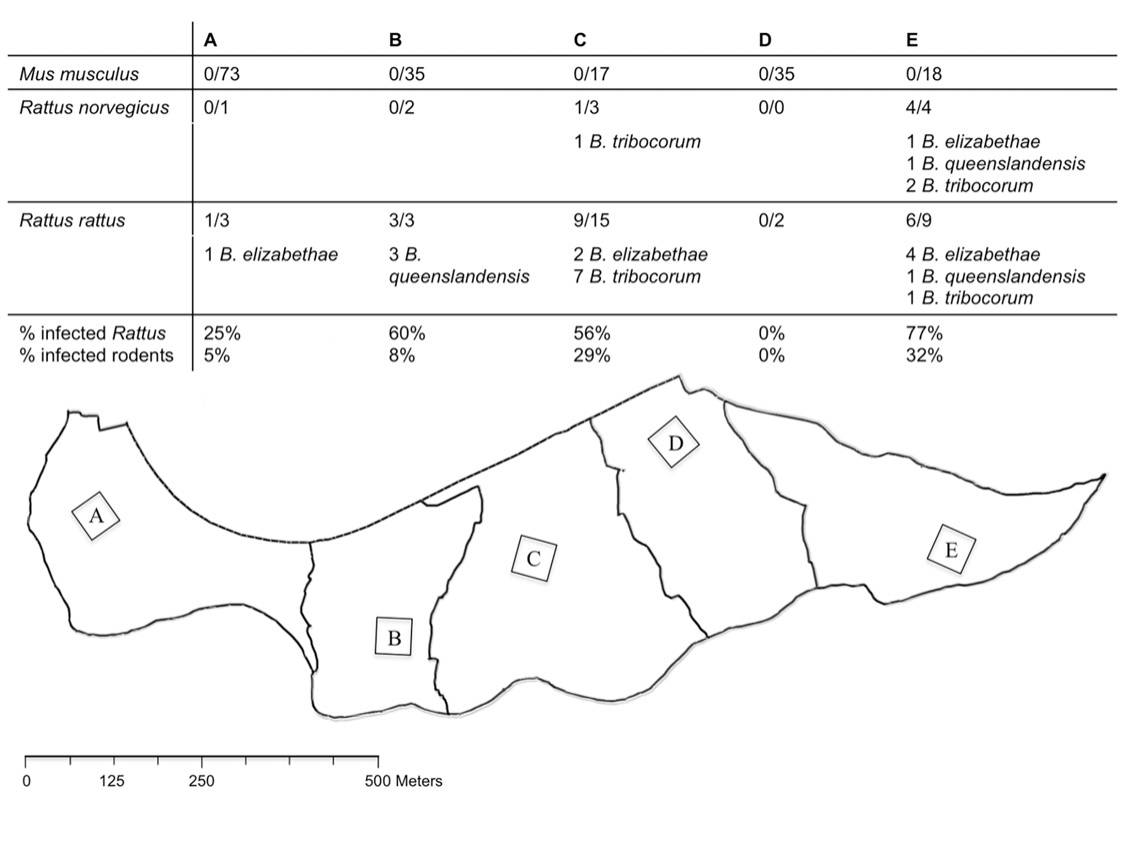
Map and summary of rodent trapping sites in Kibera. Summary of the rodents trapped and *Bartonella* isolates obtained within different trapping zones (A to E) at the Kibera study site.

In Asembo, trapping was conducted over the period of July—August 2009. Traps were placed at 50 compounds that were a randomly selected subset of livestock-owning compounds enrolled in a larger study of zoonoses epidemiology [26]. Within each selected compound, five or six medium-sized foldable Sherman traps (H.B. Sherman Traps Inc., Tallahassee, FL) were placed for three or four nights. Traps were placed in three categories of habitat: within occupied dwellings; within outbuildings, which included unoccupied dwellings, stores, latrines or kitchens separate from the main dwelling; and outside, in areas within the compound yard. In Kibera, trapping was conducted over the period of September—November 2008. The overall study site was divided for this study into five trapping zones of similar area and within each zone a 50m x 50m trapping area was defined (Fig. 2). Within each of the five trapping zones, medium-sized foldable Sherman traps were placed for a minimum of two consecutive nights and a maximum of six nights with the aim of trapping approximately 50 rodents per zone. In Kibera, all traps were placed indoors at 270 occupied dwellings.

All trapped animals from both locations were euthanized by overdose of the inhalant anesthetic halothane and whole blood was collected by cardiocentesis using aseptic technique. Blood samples were processed to remove serum and the remaining blood clots frozen at -80°C prior to testing. Blood clots were shipped on dry ice to the *Bartonella* laboratory at the Centers for Disease Control and Prevention, Fort Collins, Colorado for laboratory testing. Morphometric data were collected from each trapped animal for species identification at the National Museums of Kenya. The Asembo small mammals were submitted for archiving under accession numbers NMK 171860—NMK 171922. The Kibera rodent population included in this study is as described previously [27].

### *Bartonella* culture

Culture was performed using previously described techniques [28]. Briefly, blood clots were re-suspended 1:4 in brain heart infusion broth supplemented with 5% amphotericin B, then plated onto agar supplemented with 5% sterile rabbit blood and incubated at 35°C in an aerobic atmosphere of 5% carbon dioxide for up to 30 days. Bacterial colonies were presumptively identified as *Bartonella* based on their morphology. Subcultures of *Bartonella* colonies from the original agar plate were streaked onto secondary agar plates and incubated at the same conditions until sufficient growth was observed, usually between 5 and 7 days. Pure cultures were harvested and stored in 10% ethanol.

### DNA extraction, PCR and sequencing

The identity of presumptive *Bartonella* isolates was confirmed by PCR amplification and sequencing of a specific fragment of the *Bartonella* citrate synthase (*gltA*) gene. Crude DNA extracts were obtained from bacterial cultures by heating a heavy suspension of the microorganisms. Two oligonucleotides (BhCS.781.p and BhCS.1137.n) were used as PCR primers to generate a 379-bp amplicon of the *Bartonella gltA* gene [29]. PCR products were separated by 1.5% agarose gel electrophoresis and visualized by ethidium bromide staining. Sequencing reactions were carried out in a PTC 200 Peltier Thermal cycler (Applied Biosystems; Foster City, California) using the same primers as the initial PCR assay.

### Phylogenetic analysis

Sequences were analysed using Lasergene 12 Core Suite (DNASTAR, Madison, WI) to determine sequence consensus for the *gltA* amplicons. Unique *gltA* sequences generated through this study were submitted to GenBank (accession numbers KM233484—KM233492). The Clustal V program within the MegAlign module of Lasergene was used to compare homologous *Bartonella gltA* sequences generated in this study with others available from the GenBank database. Phylogenetic trees were constructed using the neighbor-joining method with the Kimura’s 2-parameter distance model and bootstrap calculations were carried out with 1000 replicates. *B*. *tamiae* was used as the outgroup. A criterion of >96% homology was used to define similarity of study sequences to known *Bartonella* species [30].

### Ecological analysis

Generalized linear models were used to examine associations between individual *Bartonella* infection status (culture positive or negative for *Bartonella*) and host and environmental variables in R (Version 3.0.3) [31]. Binomial family models with a logit link function were used and p values ≤ 0.05 were considered statistically significant. Variables examined included host species, sex, mass and trapping location. Data from the Asembo (S1 Table) and Kibera (S2 Table) sites were analysed separately.

### Ethics statement

Written informed consent for trapping was obtained from representatives of the study households. The protocols and consent forms were reviewed and approved by the Animal Care and Use and Ethical Review Boards of the Kenya Medical Research Institute (#1191). The study protocols were also approved by the Institutional Animal Care and Use Committee and Institutional Review Board of the U.S. Centers for Disease Control and Prevention (#5410) and complied with the Public Health Service Policy on Humane Care and Use of Laboratory Animals.

## Results

### Small mammal trapping

A total of 49 small mammals trapped at 29 compounds in Asembo and 220 rodents trapped at 143 households in Kibera were included in this study. The small mammals trapped in Asembo included *Crocidura olivieri* (n = 16, African giant shrew), and rodents of the species *Lemniscomys striatus* (n = 2, striped grass mouse), *Mastomys natalensis* (n = 14, Natal multimammate mouse), *Mus minutoides* (n = 1, pygmy mouse), and *Rattus rattus* (n = 16, black rat). All of the rodents trapped in Kibera were *Mus musculus* (n = 178, house mouse), *Rattus norvegicus* (n = 10, brown rat) or *Rattus rattus* (n = 32) (Table 1).

**Table 1:** Summary of the species and number of small mammals trapped at different locations in the two study sites that were tested for *Bartonella*.

Site	Species	Trap Location			
		**Indoor**	**Outbuildings**	**Outside**	**Location Not Recorded**
**Asembo**	*Crocidura olivieri*	5	1	10	0
	*Lemniscomys striatus*	0	0	2	0
	*Mastomys natalensis*	1	1	10	2
	*Mus minutoides*	0	0	1	0
	*Rattus rattus*	6	8	1	1
	**Total**	**12**	**10**	**24**	**3**
**Kibera**	*Mus musculus*	178	-	-	-
	*Rattus norvegicus*	10	-	-	-
	*Rattus rattus*	32	-	-	-
	**Total**	**220**	-	-	-

### *Bartonella* culture

Ten of the 49 (21%) animals trapped in Asembo were culture-positive for *Bartonella*, including: *Crocidura olivieri* (n = 1, 7%); *Lemniscomys striatus* (n = 1, 50%); *Mastomys natalensis* (n = 6, 43%); and *Rattus rattus* (n = 2, 13%). Overall, 24 of the 220 (11%) animals trapped in Kibera were culture positive: including *Rattus norvegicus* (n = 5, 50%) and *R*. *rattus* (n = 19, 60%). None of the 178 samples collected from *Mus musculus* in Kibera were positive (Table 2). Culture-positive *Rattus* species were trapped in four of the five trapping grids established at the Kibera site (Fig. 2).

**Table 2:** Summary of the number and species of the *Bartonella* isolates obtained and the prevalence of *Bartonella* infection in each population.

Site	Species	N tested	N (%) *Bartonella* positive	*Bartonella* species identified			
				*birtlesii-like*	*elizabethae*	*tribocorum*	*queenslandensis*
**Asembo**	*Crocidura olivieri*	16	1 (7%)	1	-	-	-
	*Lemniscomys striatus*	2	1 (50%)	-	-	1	-
	*Mastomys natalensis*	14	6 (43%)	-	1	5	-
	*Mus minutoides*	1	0 (0%)	-	-	-	-
	*Rattus rattus*	16	2 (13%)	-	2	-	-
**Kibera**	*Mus musculus*	178	0 (0%)	-		-	-
	*Rattus norvegicus*	10	5 (50%)	-	1	3	1
	*Rattus rattus*	32	19 (60%)	-	7	8	4

### Phylogenetic analysis

**Fig 3:**
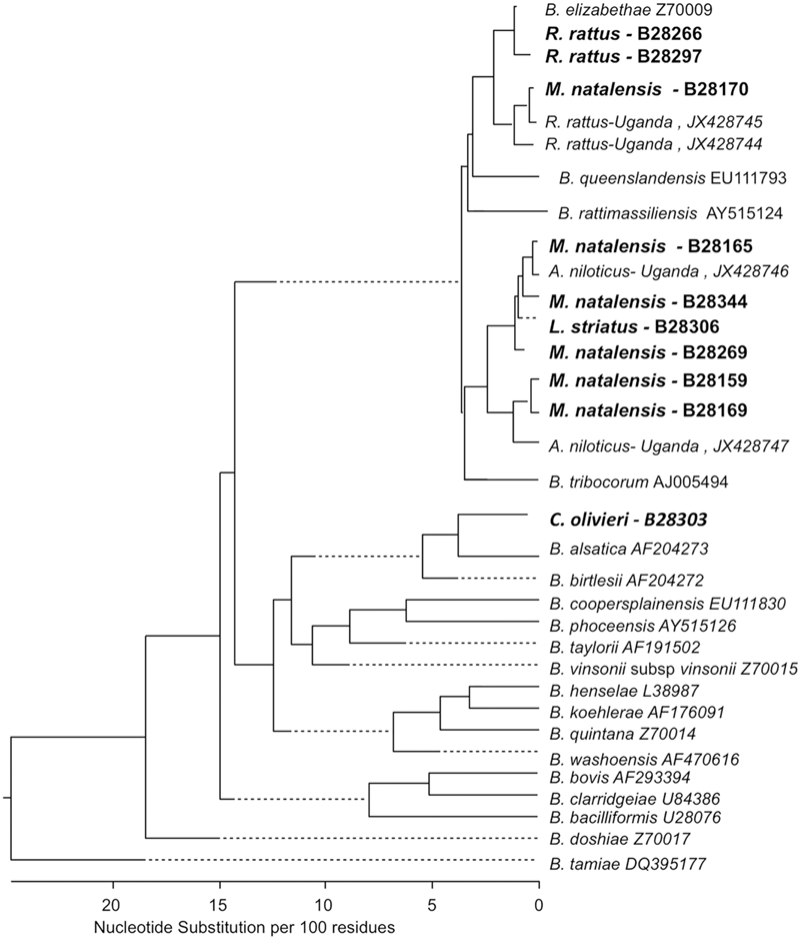
Phylogenetic tree of Asembo *Bartonella* isolates. Phylogenetic tree of *Bartonella* isolates obtained from Asembo small mammals (shown in bold) and previously described reference strains from sylvatic rodents trapped in Africa based on sequence analysis of the citrate synthase (*gltA*) gene. The phylogenetic tree was constructing using the neighbor-joining method. Dotted lines indicate negative branch lengths.

**Fig 4:**
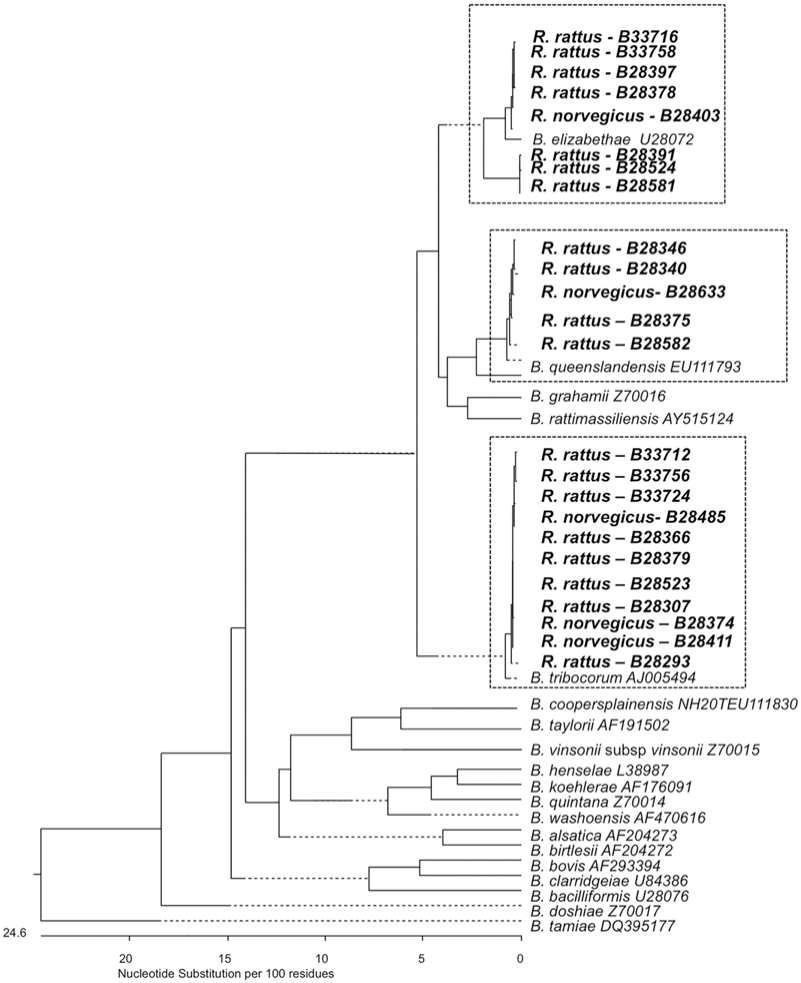
Phylogenetic tree of Kibera *Bartonella* isolates. Phylogenetic tree of *Bartonella* isolates obtained from Kibera rodents (shown in bold) and previously described reference strains for globally dispersed *Bartonella* species based on sequence analysis of the citrate synthase (*gltA*) gene. The phylogenetic tree was constructing using the neighbor-joining method. Dotted lines indicate negative branch lengths.

Information on *gltA* sequences was obtained from all 34 culture-positive animals. The phylogenetic relationships between the isolates obtained in this study and previously described *Bartonella* species are shown in Figs 3 and 4. *Bartonella* detected in one *M*. *natalensis* and two *R*. *rattus* trapped in Asembo belong to the *B*. *elizabethae* species complex based on the similarity of the *gltA* sequences (Fig. 3 & Table 2). Five additional sequences detected in *M*. *natalensis* and one detected in *L*. *striatus* were most closely related to *B*. *tribocorum*. None of the strains obtained from *M*. *natalensis* or *L*. *striatus* were identical (≥ 96% sequence identity) to reference strains of the *Bartonella* species described previously in *Rattus* species trapped elsewhere. The strain of *Bartonella* cultured from a *C*. *olivieri* was not very similar to any previously described *Bartonella* reference species but has 93.8% similarity *B*. *birtlesii*. Three pathogenic *Bartonella* species (*B*. *elizabethae*, *B*. *tribocorum* and *B*. *queenslandensis*) were detected in the two *Rattus* species sampled at the Kibera site. The *glt*A sequences for all *Bartonella* strains from Kibera rodents were identical (≥ 96% sequence identity) to reference isolates that are typical of *Bartonella* detected in *Rattus* populations globally (Table 2 & Fig. 4).

### Ecological analysis

At the Asembo location, there was a weak association between individual infection status and host species (likelihood ratio test p = 0.053) where infection probability was higher in *M*. *natalensis* individuals than in the reference species *C*. *olivieri* (OR = 11.25, 95% CI = 1.15–110.47, p = 0.038). Approximately half (24/49) of the small mammals trapped in Asembo were trapped outside (Table 1). None of the *Bartonella* positive animals trapped in Asembo were trapped within occupied dwellings. Two positive *R*. *rattus* were trapped in outbuildings but all other positive animals (one *C*. *olivieri*, one *Lemniscomys striatus* and six *M*. *natalensis*) were trapped outside. There were no statistically significant associations between the probability of *Bartonella* infection and small mammal sex or mass within the Asembo population. At the Kibera location, there was a clear influence of genus upon infection probability. None of the 178 *Mus* trapped were *Bartonella* positive but 24/42 *Rattus* were positive indicating much higher infection probability in *Rattus* (OR = Infinite). Considering the data for *Rattus* individuals only, there were no statistically significant associations between the probability of *Bartonella* infection and rodent species, sex, or mass. The proportion of infected *Rattus* and proportion of infected rodents overall varied by trapping zone in Kibera (Fig. 2). There was no statistically significant difference in the probability of *Bartonella* infection in *Rattus* from different trapping zones. However the sample size for this analysis was small and the existence of zones where no positive individuals were trapped complicate this analyses and its interpretation. Descriptively, the trapping data from Kibera fall into two groups. In zones A, B and D few *Rattus* were trapped (Fig. 2), the rodent populations in these zones were dominated by *Mus musculus* and only four *Bartonella* isolates were identified in the combined rodent populations from these three zones (Fig. 2). In contrast, in trapping zones C and E, *Rattus* made up larger proportions of the total trapped population (51% in zone C and 40% in zone E) and more *Bartonella* isolates of several species were identified in these populations.

## Discussion

This study reports isolation of *Bartonella* strains from rodent and shrew species in Asembo and Kibera, Kenya. *Bartonella* strains were found in several small mammal species with variation observed in the infection prevalence and in the strains of *Bartonella* detected between host species and study sites. The majority of *Bartonella* isolates obtained from these Kenyan mammals are genetically similar to reference strains of known human pathogens.

Several recent studies indicate that the prevalence of *Bartonella* infection in *Rattus* in Africa may be low in contrast to the frequently high prevalences observed in Asian *Rattus* populations [18–20,32]. It has been argued that this pattern of lower prevalence in African *Rattus* populations could be attributed to host escape during colonization [19,20], a phenomenon where relatively small founding populations of invading species can leave their parasites behind when colonizing new areas [33]. Consistent with this, a relatively low prevalence was seen in *R*. *rattus* from Asembo (13%). However, the high infection prevalence observed in *Rattus* trapped at the Kibera site (e.g. 50% *R*. *norvegicus* and 60% *R*. *rattus*) is more similar to prevalence values observed in studies of Asian *Rattus* populations than to other African populations [19]. There are multiple possible explanations for the difference in the prevalence observed in *Rattus* at these two sites. Phylogenetic analyses indicate that *B*. *elizabethae* complex strains originated in Southeast Asia and have been disseminated throughout Asia, Europe, Africa, Australia and the Americas through multiple dispersal events of commensal *Rattus* species [5]. The Kibera study site is near the centre of Nairobi, the Kenyan capital, and is likely to have greater international connectivity (in terms of international rodent movement through trade etc.) than the Asembo site, which is more rural. The higher prevalence observed at the Kibera site could therefore be explained by repeated introduction of *Rattus* and their associated *Bartonella* species to this site [19,33]. Further analyses would however be needed to elucidate the colonization history of *Rattus* and their associated *Bartonella* at these sites specifically. The number of species trapped in Kibera was smaller than the number trapped in Asembo, indicating a simpler species composition at this site and these data could also suggest a possible dilution effect of the increased community complexity in Asembo on *Bartonella* prevalence [34]. Finally, temporal dynamics in host and ectoparasite population structure are known to affect *Bartonella* infection prevalence [21,35]. This study involved cross-sectional trapping surveys conducted at different times of year in the two study locations. There are few data on the seasonal variation in the abundance or diversity of the rodent populations at these sites but it is likely that there are seasonal influences upon rodent abundance and diversity with differences between the urban Kibera site and the more rural Asembo site in the seasonal population dynamics observed [36]. It is therefore possible that differences in the sampling time may have contributed to the differences in infection prevalence seen between these two *Rattus* populations.

Notably, no bartonellae were detected in *Mus musculus* trapped in Kibera despite a large number of tested animals and high infection prevalence observed in *Rattus* trapped in the same locations. Low-level *Bartonella* infection has been reported from *Mus* trapped in Ethiopia but the absence of *Bartonella* in *Mus* was also reported in a small-scale study from Nigeria [19,37].

All of the Kibera rodents were trapped within residents’ homes. In contrast, although nearly half of the animals trapped in Asembo were trapped indoors or in outbuildings, none of the positive animals at this site were trapped indoors, and only two positive animals were trapped in outbuildings. Approximately one third of the animals trapped outside in Asembo however were culture positive for *Bartonella*. The two culture positive animals trapped in outbuildings were *R*. *rattus* and they were carrying *Bartonella* similar to the *B*. *elizabethae* reference strain (Fig. 2). This species was most commonly found indoors or in outbuildings (14/15 records) and therefore may pose a risk due to closer human contact, even though only 2/16 were positive.

Many of the *Bartonella* detected at in this study (except the birtlesii-like isolates from *Crocidura*) belong to the *Bartonella elizabethae* complex and many of the strains identified in invasive *Rattus* hosts particularly are closely related to known human pathogens. All of the *Bartonella* strains isolated from Kibera rodents have ≥ 96% sequence identity with strains that are common in *Rattus* species sampled in Asia and on several other continents [5]. In contrast, several strains isolated from Asembo rodents and shrews were less similar to the international reference strains from *Rattus* and were more similar to isolates gathered previously from Ugandan rodents, suggesting a longer history of circulation of these strains within these species at the Asembo site. The identification of similar *B*. *tribocorum* sequences in *Mastomys* and *Lemniscomys* individuals trapped in Asembo suggests an absence of strong host-pathogen associations in these populations.

There is a high incidence of acute febrile illness in people in both Asembo and Kibera [25]. A variety of pathogens are known to account for a proportion of febrile illness in Asembo and Kibera but considerable proportions remain unexplained [38–41]. *Bartonella* species have been identified as important causes of human febrile illness in several global settings but there has been little investigation of the impact of bartonellosis upon human health in Africa particularly and it is conceivable that *Bartonella* may be an important cause of febrile illness in these study populations.

The data presented from the Asembo and Kibera sites indicate clear differences in: the prevalence of *Bartonella* infection in the same host (*Rattus* species) at the two sites; the prevalence of infection in different hosts trapped at the same sites; the abundance of different infected hosts between the two locations and also between trapping zones in Kibera; the strains of *Bartonella* detected and finally in the locations within communities where rodents overall and *Bartonella* infected rodents were trapped. The impact of this variation in rodent host community composition, infection prevalence, ectoparasite vector preferences, and other ecological factors need to be understood to evaluate human *Bartonella* infection risks at these sites. The data presented here suggest that investigations of the multi-host infection dynamics of *Bartonella* and the public health significance of *Bartonella* infections at these Kenyan locations and others where there are close associations between people and small mammals are warranted.

## Supporting Information

S1 Table Dataset for Asembo analyses. (XLS)

S2 Table Dataset for Kibera analyses. (XLSX)
